# Effects of Dietary Forage Proportion on Feed Intake, Growth Performance, Nutrient Digestibility, and Enteric Methane Emissions of Holstein Heifers at Various Growth Stages

**DOI:** 10.3390/ani9100725

**Published:** 2019-09-26

**Authors:** Lifeng Dong, Binchang Li, Qiyu Diao

**Affiliations:** Feed Research Institute, Chinese Academy of Agricultural Sciences/Beijing Key Laboratory for Dairy Cow Nutrition/Key Laboratory of Feed Biotechnology, Ministry of Agriculture/Sino-US Joint Lab on Nutrition and Metabolism of Ruminant, Beijing 100081, China or l18394151270@163.com (B.L.)

**Keywords:** methane, heifer, forage-to-concentrate ratio, prediction equation, sulphur hexafluoride tracer technique

## Abstract

**Simple Summary:**

Enteric methane (CH_4_) emission from ruminants is a large source of anthropogenic greenhouse gas production, which is an inevitable by-product when feedstuff is digested and fermented in the rumen, representing approximately 7% of dietary energy loss. Although the Chinese government has committed to reduce CH_4_ emissions under the requirement of the Copenhagen Accord (2009), there is lack of accurate CH_4_ emission data from young cows as the guideline of IPCC gives little consideration to the variations of geographic conditions, animal physiology stages, and dietary components of dairy production system. Our study investigated the effects of different dietary forage-to-concentrate on feed intake, growth performance, nutrient digestibility, and enteric CH_4_ emissions of Holstein heifers under various growth stage, and developed the prediction equations using production and emission data. Our results demonstrated that enteric CH_4_ emission was significantly affected by dietary composition and physiological condition; results obtained from the current study will be of great importance for development of regional or national emission inventories and mitigation approaches for heifers at specific growth stage.

**Abstract:**

Enteric methane (CH_4_) emissions from young ruminants contribute to a substantial proportion of atmospheric CH_4_ accumulation. Development of emission inventory and mitigation approaches needs accurate estimation of individual emission from animals under various physiological conditions and production systems. This research investigated the effect of different dietary concentrate contents on feed intake, growth performance, nutrient digestibility and CH_4_ emissions of heifers at various stages, and also developed linear or non-linear prediction equations using data measured by sulphur hexafluoride tracer technique. Increasing dietary concentrate contents increased feed intake and growth rate, enhanced nutrient digestibility, and reduced enteric CH_4_ emissions. Heifers at the age of 9, 12, and 15 months with an average weight of 267.7, 342.1, and 418.6 kg produced 105.2, 137.4, and 209.4 g/day of CH_4_, and have an average value of CH_4_ energy per gross energy intake (Y_m_) 0.054, 0.064, 0.0667, respectively. Equations relating CH_4_ emission values with animal and feed characteristics were developed with high determination coefficients for heifers at different growth stages. Dietary concentrate contents had significant influence on overall performance of heifers. These data can be used to develop regional or national emission inventories and mitigation approaches for heifers under various production regimes in China.

## 1. Introduction

Enteric methane (CH_4_) is a final product of ruminal fermentation via methanogenesis, which contributes substantially to atmospheric CH_4_ accumulation. As dietary structural carbohydrates (e.g., cellulose and hemicellulose) are degraded by ruminal microorganisms, CH_4_ emission represents up to 12% loss of dietary energy ingested in the rumen [1]. Thus, reducing enteric CH_4_ emissions will help improve energy utilization efficiency and alleviate environmental pressures for dairy production regimes. According to the national report, CH_4_ emissions in 2005 from agriculture sector accounts for 25.5 Tg, of which approximately 57% is from rumen fermentation within ruminant production system in China [2]. As dairy population and milk production increased by 24 and 58 times, respectively, from 1961 to 2010, it is projected that the total CH_4_ emissions in 2030 will reach 52.1 Tg [3]. Meanwhile, in response to the domestic and international pressures on sustainable development, the Chinese government has made the commitment to reduce greenhouse gases (GHG), which has been incorporated in to the 2009 Copenhagen Accord [4]. Although Tier-2 methodology from International Panel on Climate Change (IPCC) guidelines is commonly used in many countries for the quantification of CH_4_ emission inventories [5], it gives little consideration to the variations of geographic conditions, animal physiology stages, and dietary components [6,7]. In addition, this methodology tries to calculate the CH_4_ emission for the whole dairy population using a default value derived from lactating cows. In China, approximately 60% of the dairy population is milking cows, and the remainder is heifers (i.e., 5.68 million of heifers) [8]. Different physiological conditions and varied composition and abundance of ruminal methanogens demonstrated great difference of CH_4_ emission of lactating cow and heifer, indicating the importance of quantifying the individual emissions for these animals [3]. However, limited studies have examined the effects of dietary components on CH_4_ emissions of heifers under different physiological conditions. Therefore, the objective of the current study is to assess the effect of different dietary concentrate contents on the enteric CH_4_ emissions of Holstein heifers at various stage, and develop prediction equations using data collected using sulphur hexafluoride (SF_6_) tracer technique. 

## 2. Materials and Methods 

### 2.1. Animals, Experimental Design, and Diets 

This study was conducted in 2018 at the Zhongjiayonghong dairy farm located in Fangshan district, (Beijing, China, latitude: N39°39’6’’ and longitude: E116°12’21’’). Forty-five Chinese Holstein heifers with an initial body weight (BW) of 264.9 ± 25.6 kg (mean ± SD) were used in this study with three measurements taken at age of 9, 12, and 15 months, respectively. In each experiment stage, heifers were balanced by date of birth, age and BW and offered randomly assigned to 1 of 3 treatments (n = 15) in which animals were individually offered diets containing 30, 40, and 50% of concentrate (C30, C40, and C50, respectively). Each experimental period was 32 days in length, including 18 days for adaptation, followed by 8 days for gas measurement and 6 days for nutrient digestibility. Heifers were housed individually with free access to feed and water throughout the whole experiment. All animal care and handling procedures were reviewed and approved by the Animal Ethics Committee of Chinese Academy of Agricultural Sciences (protocol number 019–2018) prior to the start of the experiment. 

In period 1, cows received their diet as a total mixed rations (TMR) that composted of corn silage, wildrye, and a typical ration of concentrate on Chinese commercial farms. In period 2 and 3, alfalfa was included in the diet based on the ration of period 1 (Table 1). The TMR were prepared daily using a feed mixer (Belle Engineering Ltd., Derbyshire, UK) and distributed *ad libitum* (5% refusals, on an as-fed basis). All diets in three periods were formulated to meet the recommendation of Ministry of Agriculture of P. R. China. For all of the three periods, cows were fed twice daily between 0600 and 0800 h, and 1600 and 1800 h. Feed refusals were collected and weighted to determine the daily feed intake.

### 2.2. Enteric Methane Emission Measurement 

Enteric CH_4_ emissions were measured from individual cows using the SF_6_ tracer technique with minor modification of Deighton et al. [9]. Generally, empty permeation tubes were filled with 450 mL of 99.999% pure SF_6_ by immersing in liquid nitrogen. The release rate was determined by incubating the permeation tubes in an oven at 39 °C and weighting each one twice a week for 4 weeks. The calculated releasing rate of the SF_6_ tubes ranged from 3.13 to 3.84 (mean, 3.28 ± 0.175) mg /day in period 1, from 3.10 to 3.70 (3.32 ± 0.266) mg/day in period 2, and from 3.10 to 3.60 (3.20 ± 0.167) mg/day in period 3, respectively. Each cow was randomly administrated with one SF_6_ tube using a balling gun three weeks before the commence of the experiment. 

A back-mounted harness was used to support the canister (volume = 1.85 L) for continuously sample collection; the canisters were washed by flushing 99.999% pure nitrogen and evacuated to over 98 kPa vacuum. The sampling rate of canister was approximately 0.25 mL/min by crimping a stainless-steel capillary tube within the sampling tubing. Canisters were removed after 24 h and residual vacuum was recorded before addition of nitrogen gas. Background gas samples of SF_6_ and CH_4_ were also collected daily by using six additional canisters that were either placed on the back of animals or about 2.0 m above ground level of the experimental barn.

Gas samples were analyzed using a gas chromatography system (GC126, Shanghai Precision Instruments Co., Ltd., Shanghai, China) equipped with a flame-ionization detector (FID) and an electron-capture detector (ECD). The ECD operated at 300 °C with a molecular sieve 0.5 nm column and the FID at 150 °C with a Porapack N 80–100 mesh column (Shanghai Precision Instruments Co., Ltd., Shanghai, China) for determination of SF_6_ and CH_4_, respectively. Ultra-high purity nitrogen gas (99.999%) was used as carrier gas at 40 mL/min flow and analysis was performed after calibration with standard gases for SF_6_ and CH_4_. The daily CH_4_ emission was calculated as follows:

CH_4_ = SF_6_ × [(CH_4*sample*_ − CH_4*background*_)/(SF_6*sample*_ − SF_6*background*_)] × (16/146) × 1000

where CH_4_ is the calculated emission (g/d); SF_6_ is the measured releasing rate of each SF_6_ permeation tube (mg/day); the concentration of CH_4_ sample and CH_4_ background are expressed in ppm and concentration of SF_6_ sample and SF_6_ background in ppt; 6 and 146 are the molecular mass (g/mol) of CH_4_ and SF_6_, respectively; the factor of 1,000 is used to calculate CH_4_ in units of g/day. 

### 2.3. Nutrient Digestibility and Laboratory Analyses 

During the last 6 d of each experimental period (digestibility experiment), 5 cows out of each treatment were moved to metabolic stalls for nutrient digestibility measurement using a modified method of acid-insoluble ash (AIA) [10]. Generally, rectal feces were collected from the rectum to obtain representative samples (day 1: 1000 and 2200 h; day 2: 0200 and 1400 h; day 3: 0500 and 1700 h; day 4: 0800 and 2000 h; day 5: 1100 and 2300 h; day 6: 0600 and 1800 h). Fresh samples over the 6 days period from each cow were composited and analyzed by using 2N HCl. The equation used to calculate digestibility was as follows:

Nutrient digestibility = 100 − [100 × (ADIA in DM consumed,%/ADIA in feces, %)/(nutrient in feces, %/nutrient in consumed DM, %)]

in which ADIA = acid detergent insoluble ash.

Representative feed samples were collected during adaptation and experimental period for chemical composition determinations. Dietary gross energy (GE) content was determined by bomb calorimetry (1108 Oxygen bomb, Parr Instruments, Moline, IL, USA). Dry matter, neutral detergent fiber (NDF), acid detergent fiber (ADF), crude fat, and ash were determined using AOAC International (2006), and crude protein (CP) was measured using combustion analyzer (Leco FP-528 N, Fullerton, CA, USA).

### 2.4. Statistical Analyses

The effect of dietary concentration levels on growth performance, nutrient digestibility and enteric CH_4_ emissions was evaluated using two analytical approaches as described by Dong et al. [1]. Generally, the ANOVA procedure was used with the three treatments fitted as a fixed effect and animals within each treatment fitted as random effects during the analysis. Other necessary variables such as initial BW and date of birth were fitted as covariates, when appropriate, for evaluation of enteric CH_4_ emissions. Prediction equations were developed using restricted maximum likelihood model as treatments were fitted as a fixed effect. Significant effects were noted at *p* < 0.05. The statistical program used in the current study was Genstat 14.2 (14th edition; Lawes Agricultural Trust, Rothamsted, UK).

## 3. Results

### 3.1. Effects on Nutrient Intake and Growth Performance

Dietary ingredients and chemical composition are presented in Table 1. The diets are planned to differ in concentrate contents and feed analysis indicated that NDF and ADF decreased with increasing concentrate feed contents in any of periods one to three. Accordingly, the opposite happened with the NFC fraction of the diets.

Nutrients and energy intake and growth performance are presented in Table 2. Overall, DM, OM, NFC, and GE intake increased with increasing concentrate contents in any of periods one to three (*p* < 0.05); however, these values did not differ significantly between the C40 and C50 treatments in period one or between C30 and C40 treatments in period two (*p* > 0.05). Dietary NDF intake was similar among the three treatments in period one (2.12 vs. 2.21 vs. 2.17 for C30, C40 and C50 treatment, respectively, *p* > 0.05), whereas heifers in C30 treatments consumed more NDF than the other two treatments in periods two and three (*p* < 0.05). Weight gain increased with increasing concentrate feed contents in the diet (*p* < 0.05) with an average ADG value of 1.26, 1.16, and 0.97 kg/day for heifers in periods one to three, respectively.

### 3.2. Effects on Apparent Nutrient Digestibility

Apparent nutrient digestibility data are presented in Table 3. Overall, CP and NDF digestibility increased with increasing concentrate feed contents throughout the three experimental periods (*p* < 0.05). However, DM and OM digestibility remained similar among the three treatments in period one, whereas both values increased linearly as concentrate increased in periods two and three (*p* < 0.05). Dietary ADF digestibility was unaffected by different concentrate contents in period two with a value of 68.4, 69.4 and 71.1 for C30, C40 and C50 treatments, respectively (*p* > 0.05). Moreover, there was an increasing trend in digestibility of average DM (74.47 vs. 75.50 vs. 78.47), OM (76.43 vs. 79.20 vs. 80.50) and ADF (68.42 vs. 69.63 vs. 74.00) for period one to three, respectively.

### 3.3. Effects on Enteric CH_4_ Emission

Enteric CH_4_ emission data of each experiment period are presented in Table 4. Daily CH_4_ production and CH_4_-E were significantly affected by treatments that both sets of parameters decreased linearly with increasing concentrate feed contents in the diets in any periods of one to three (*p* < 0.05). 

Individual CH_4_ intensity including CH_4_/DM intake, CH_4_/OM intake, and CH_4_/NDF intake decreased linearly with increasing dietary concentrate feed contents throughout the three experiment periods, whereas no difference was observed for CH_4_/NDF intake between C40 and C50 (55.81 vs. 54.60 g/kg) treatments in period two (*p* > 0.05). CH_4_-E per gross energy intake (Y_m_) decreased significantly (*p* < 0.05) with increasing concentrate contents in any periods of one to three. Furthermore, although comparison of the effect of experimental periods on CH_4_ emissions was the objective of this study, the average of Y_m_ value was 0.0564, 0.0639, and 0.0667 in periods one to three, respectively.

### 3.4. Development of Prediction Equations

Prediction equations of CH_4_ emissions in each period are presented in Table 4 and Table 5. Growth and feed intake parameters were used to develop these relationships, which were significantly correlated (*p* < 0.01) with coefficient of determination values ranging from 0.27 to 0.74. 

**Figure 1:**
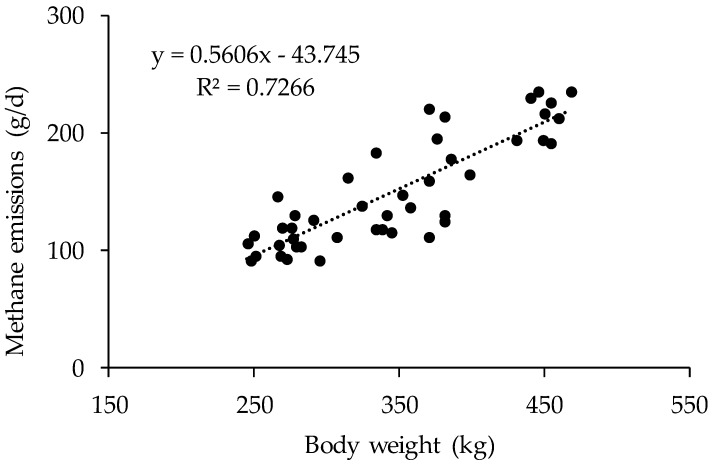
Linear relationship between body weight and enteric methane emissions of Holstein heifers.

**Figure 2:**
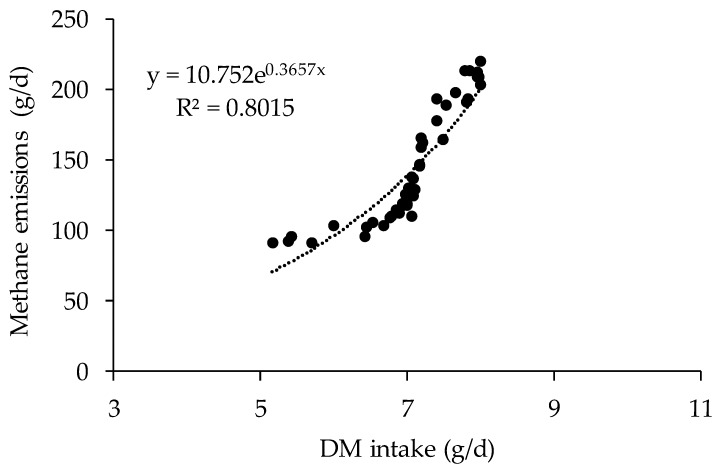
Non-linear relationship between dry matter intake and enteric methane emissions of Holstein heifers.

Overall, relationships obtained in period three had highest values of determination values when compared with those from the other two periods. The strongest relationship was observed between CH_4_ emission and DM intake in period three (Equation (8) in Table 5, R^2^ = 0.74), whereas CH_4_ production was relatively poor related with BW for heifers in period 1 (Equation (1) in Table 5, R^2^ = 0.47). Furthermore, emissions data derived from all three periods were pooled to develop overall CH_4_ prediction equations (Figure 1 and Figure 2). Feed intake and BW were significantly correlated with CH_4_ emission (*p* < 0.01) and coefficient of determination value was 0.727 and 0.802 for linear and non-linear equations, respectively. Furthermore, a range of linear and non-linear prediction models were developed using the whole data sets of animal production and feed intake values (Equations (10) to (20), Table 6). Generally, improved values of R^2^ can be observed with more variables were incorporated in to the models. For example, highest value of R^2^ of 0.820 was observed for the models relating CH_4_-E to BW, DM intake, and NFC intake (*p* < 0.01), whereas a relatively low value of R^2^ of 0.593 was observed for Equation (12), which relates CH_4_-E to DM intake and NDF intake (*p* < 0.01). However, there was no such trends for non-linear models as highest value of R^2^ was observed for the relationship between CH_4_-E and NDF intake (Equation (18)).

## 4. Discussion

### 4.1. Effects on Feed Intake and Growth Performance

A number of studies have demonstrated that increasing dietary concentrate contents would increase feed intake of heifers, although they are typically fed high-fiber diets due to physiological and economic considerations [11]. In accordance with the previous studies, moving from 2.09 to 3.59 kg/day and 2.23 to 3.98 kg/day from of concentrate treatments increased DM intake by 0.20 and 0.52 kg/day in period two and three, respectively. Aguerre at al. [12] reported a significant increase of NDF intake from 5.4 to 6.5 kg/day as dietary forage-to-concentrate increased from 47:53 to 68:32. However, although only a numerical change was observed in NDF intake, NFC intake increased significantly as concentrate contents increased from 30 to 50% in period one. In the current study, alfalfa was introduced into the diets in the last two periods. This high-quality forage would be responsible for the significant increase of feed intake for the C50 treatments due to its good palatability and high level of digestibility [13]. Consequently, increased ADG were achieved in the current study as a direct result of higher energy density of the higher concentrate diets and increased feed intake [14].

### 4.2. Effects on Apparent Nutrient Digestibility

Generally, nutrient digestibility increased with increased concentrate supplementation for heifers in any periods of the present study. Moody et al. [15] reported that increasing dietary corn silage contents reduced DM digestibility of Holstein heifers either at the age of 6 or 12 months. Jiao et al. [16] found similar digestibility values of DM, NDF and ADF to our results for heifers at various ages. Different from our findings, Moody et al. [15] reported that NDF digestibility decreased as concentrate proportion increased in the diet, and concluded that this significant reduction of NDF digestibility may be due to the variations of passage of different forage in the diet and growth condition of animals [17]. However, as corn silage was commonly used in each experimental period, only alfalfa was introduced into the period two and three of the present study. This inclusion of different forage types might explain different nutrient digestibility among the three treatments in any period of one to three. Sarwar et al. [18] reported little difference of nitrogen digestibility for Holstein cows fed diets varying in proportion of NDF. Recent studies conducted by Drewnoski and Poore [19] and Trotta et al. [20] found that increasing dietary concentrate level increased the total tract CP digestibility from 53.1 to 58.1% for beef cattle. The current study showed that there was a positive relationship between dietary concentrate level and CP digestibility, which had similar trends to the digestibility values of other nutrients. Nousiainene et al. [21] suggested that increased CP digestibility was associated with improved diet digestibility, which may be resulted from increased dietary CP concentrate and a dilution of metabolic and endogenous fecal nitrogen. However, these authors also suggested that the amount of dietary CP concentration instead of amount of concentrate was related to changes of dietary CP digestibility. Dietary CP content increased from 15.7 to 18.7% (period 1) or from 14.1 to 14.7% (period 2) with concentrate level increasing from 30 to 50% in the present study. However, it still needs further study to elucidate the direct relationship between nutrient digestibility and dietary composition as some cofounding factors need to be considered during analysis. However, it is worth noting that the nutrient digestibility values in the present study were obtained using acid-insoluble ash as internal marker, as this method has been extensively recognized for determination of diet digestibility due to reliable digestibility estimates [22,23]. Previously, the standard procedure for measuring total-tract apparent digestibility involved total collection of feces and urine, whereas alternative approaches including acid-insoluble ash and indigestible NDF were proposed and used as they required a small number of animals and produced accurate results [24,25]. Nevertheless, nutrient digestibility values obtained in the present study were consistent with the previous studies, which indicated that acid-insoluble ash method may be a suitable and convenient method although the total collection should still be considered the best choice [26]. 

### 4.3. Effects on Enteric Methane Emissions

Due to the large population of China’s dairy industries, it is becoming increasingly important to quantify CH_4_ emissions for cows at different ages and under various production systems. However, until recently, several studies investigated the effects of dietary concentrate contents on CH_4_ emissions for heifers. Boland et al. [27] reported similar CH_4_ emissions (121 vs. 132 g/day) for grazing beef heifer that consumed different herbage masses. Jiao et al. [16] examined CH_4_ emissions from heifer and steer at various growth stage, and reported an average daily CH_4_ emission of 93.5, 159.5, 175.0, and 188.5 g/day for young stock at the age of 6, 12, 18, and 22 months under confined condition. These values are similar to the recent study of Morrison et al. [28] who measured CH_4_ emissions from grazing heifers using the SF_6_ tracer technique. However, emission data for heifers at the age of 15 months were higher than those for confined heifer and steer or for young stock in grazing condition [28]. 

As enteric CH_4_ emission represents the final production of ruminal fermentation via methanogenesis, it can be significantly affected by a range of factors including animal physiological state, dietary components, and measurement technique. Increased concentrate proportion resulted in reduced CH_4_ emissions in the current study. These values were consistent with studies of Muñoz et al. [29], who also decreased CH_4_ production with increasing concentrate level up to 6 and 5 kg of concentrate per day, respectively. Generally, inclusion of high level of concentrate in the diet represents higher content of readily fermentable substance (e.g., starch) than that of high forage diets. Previous studies demonstrated that starch-rich diets reduced ruminal pH and H_2_ concentration, and shifted fermentation patter towards to an increased propionate formation, which would depress the activity of methanogens and consequently reduce CH_4_ emissions [30,31]. Moreover, the composition and structure of ruminal methanogens was demonstrated to differ across heifer physiological stages, which would affect the enteric CH_4_ emissions of heifers [32]. Although the main objective of the current was not to examine the archaeal community in the rumen, results showed an increasing trend in CH_4_ emissions as the growth of heifer advanced, which would reflect the changes and distribution of ruminal methanogens. 

Regional or national enteric CH_4_ emission inventories in many countries are currently estimated using the Tier-2 methodology from International Panel on Climate Change (IPCC) guidelines. As with Tier-2 approaches, default prediction values for Y_m_ from adult dairy cows in the 1997 (0.060) [33] and 2006 (0.065) [5] IPCC guidelines were recommended for the CH_4_ estimation of the whole dairy population. However, adoption of a default and fixed value has becoming a major concern because it can vary considerably with varying geographic conditions, cow breed and physiological stages, and dietary characteristics [6,7]. Boadi et al. [34] reported an Y_m_ value of 0.067 or 0.076 for yearling heifers either fed ad-libitum or under restricted feeding condition. Morrison et al. [28] calculated Y_m_ values for calves, yearling heifer, and in-calf heifer with an average age of 8.5, 14.5 and 20.5 months, and found that the calculated Y_m_ was 0.057, 0.0675, 0.059 for each period. In accordance with those results from confined lactating cows or heifers at pasture, a range of Y_m_ values between 0.0454 and 0.0769 were obtained across all heifer ages in the current study, which lied within the range (0.036–0.114) obtained under diverse production systems [35]. Therefore, these prediction factors achieved on a regional production basis in China can be used and improve the prediction accuracy for cows at specific developmental stages. Furthermore, variations of Y_m_ values were also examined when heifers were fed different concentrate contents in the diet. As report previously, variations of dietary components such as starch: NDF ratios can change the rumen fermentation environment and methanogenesis functions, which consequently affect the CH_4_ emissions. In the present study, Y_m_ values decreased from 0.0686 to 0.0454, 0.0742 to 0.0558, 0.0769 to 0.0568 when concentrate intake increased from 1.68 to 3.45, 2.09 to 3.59, 2.23 to 3.98 kg/day in period 1 to 3, respectively. These results are consistent with the grazing studies of van Wyngaard et al. [35], who reported that Y_m_ of lactating Jersey cows significantly decreased from 0.0891 to 0.0785 when the concentrate increased from 0 to 8 kg/day.

### 4.4. Prediction Equations for Enteric Methane Emissions

Enteric CH_4_ emission predictions have been widely developed based on mathematical or statistical association of nutrient intake, dietary nutrient composition and digestibility and other animal factors with enteric CH_4_ emissions [36]. In agreement with previous studies, DM and GE intake were the best predictors of CH_4_ emissions in this study with values of R^2^ ranging from 0.67 to 0.74. Similar R^2^ values of 0.68 with DM intake and 0.70 with GE intake were reported for beef cattle measured using respiration calorimeters [37]. Appuhamy et al. [7] evaluated performance of more than 40 empirical models in predicting enteric CH_4_ emissions, and suggested that DM intake alone may be sufficient to achieve satisfactory prediction accuracy inventory purposes [38]. A meta-analysis conducted by Charmley et al. [39] showed that a large data set including both dairy and beef cattle can significantly enhance the relationship between DM intake and CH_4_ emissions, with a high value of determination coefficient and an intercept close to zero when DM intake ranged from 2 to 28 kg/day. The data was pooled together and a linear or nonlinear relationship was observed between BW, DM intake and CH_4_ emissions in the current study. However, curvilinear relationship between DM intake and CH_4_ production was observed when dairy cows were fed relatively high proportion of concentrate [40]. It was suggested that linear relationship between DM intake and CH_4_ production can be achieved when the concentrate level was below 30% [39]. Yan et al. [37] reported that the coefficient of determination for the relationship between DM intake and CH_4_ emissions were highly affected by several factors including growth stage, dietary concentrations of protein and carbohydrate fractions. For example, coefficients for DM intake increased from 24.21 to 51.72 for heifers at the age of 9 to 15 months, although this difference did not reach significance. Moreover, lower values of R^2^ from 0.42 to 0.47 were observed when animal characteristic such as BW was used as a single predictor variable. Jiao et al. [16] reported an increase of 0.252 kg/day CH_4_ for an increase of 1 kg of heifer BW, which was similar to our findings that an average increase of 0.203 kg/day CH_4_ was observed for each unit increase of BW.

Although linear models can be mathematically developed using dietary intake and composition variables, enteric CH_4_ emissions may not follow a linear trend as generation of CH_4_ can be affected by ruminal function and fermentation dynamics. Among the non-linear models developed using the whole data sets of the present study, a highest R^2^ value of 0.82 was observed when BW, DM intake and NFC intake were incorporate in to the equation. However, a range of relative lower values of R^2^ were also found for those exponential or power equations. This result was in consistent with the previous research of Mills et al. [41] and Patra et al. [42], who found minor difference in RMSE percentage between the linear and non-linear models. Although non-linear models required more variables to obtained the accurate methane emissions results, Mills et al. [41] suggested that non-linear models would be better for quantifying CH_4_ production in a wide range of production variables; especially, as they could be more appropriate when extreme values were obtained during the practical application [42]. The slopes of dietary DM and NDF intake were positively related to enteric CH_4_ emissions, whereas increasing dietary NFC intake may reduce CH_4_ emission. Diets rich in non-structural carbohydrates such as starch and sugars are converted to propionate in the rumen with less hydrogen and CH_4_ production. However, fermentation of fibrous materials would favour the formation of acetate and butyrate, which would have positive impact on CH_4_ emissions. 

## 5. Conclusions

It is concluded that increasing dietary concentrate contents improves feed intake and growth performance, and nutrient digestibility. Enteric methane emissions decrease significantly with increasing concentrate contents. A range of CH_4_ conversion factors are derived from the current study, reflecting the variations of animal and dietary characteristics under the typical production regimes in China. Together with prediction equations, these data will be of great importance for development of regional or national emission inventories and mitigation approaches for heifers at specific growth stage.

## Figures and Tables

**Table 1:** Ingredient and chemical composition of diets in the current study.

Item		Period 1 (9 months)			Periods 2 and 3 (12 and 15 months)		
		C30	C40	C50	C30	C40	C50
Ingredient							
	Corn silage	42	36	30	42	36	30
	Chinese wildrye hay	28	24	20	14	12	10
	Alfalfa	-	-	-	14	12	10
	Concentrate	30	40	50	30	40	50
Nutrient, DM basis							
	Dry matter	93.6	93.5	93.6	93.9	93.7	93.3
	Organic matter, %	91.8	91.7	91.2	93.1	92.8	92.2
	Gross energy, MJ kg^-1^	18.0	18.1	18.1	16.8	16.7	16.6
	Crude protein, %	15.7	17.8	18.7	14.1	14.5	14.7
	Ether extract, %	4.1	4.0	4.0	3.7	3.8	3.8
	Ash, %	8.2	8.3	8.8	6.9	7.2	7.8
	Neutral detergent fiber, %	37.8	34.2	31.4	36.8	32.6	29.3
	Acid detergent fiber, %	15.6	14.0	12.0	19.8	17.5	14.8
	Ca, %	0.5	0.5	0.5	0.3	0.5	0.6
	P, %	0.2	0.3	0.3	0.2	0.2	0.3

C30 = diet containing 30% of concentrate, C40 = diet containing 40% of concentrate, C50 = diet containing 50% of concentrate; Concentrates were purchased from a commercial company (Beijing Sanyuan Breeding Technology Corporation, Beijing, China), which mainly comprise corn, wheat bran, soybean meal, calcium hydrophosphate, limestone and salt. The nutrient content is: crude protein ≥17%, ether extract ≥2.5%, crude fiber ≤9.0%, Ca = 0.5–1.5%, P = 0.4–1.0%, NaCl = 0.5–2.0%, Lysine ≥0.6%.

**Table 2:** Effects of different dietary concentrate levels on the growth performance of Holstein heifers at age of 9, 12 and 15 months.

Item		Treatments			SEM	*p*-Value
		C30	C40	C50		
9 months						
	Age, month	9.5	9.5	9.4	1.46	0.987
	BW, kg	246.2^b^	274.3^a^	282.7^a^	5.16	0.046
	DM intake, kg/day	5.61^b^	6.47^a^	6.90^a^	0.164	<0.01
	OM intake, kg/day	5.15^b^	5.94^a^	6.30^a^	0.152	0.007
	NDF intake, kg/day	2.12	2.21	2.17	0.093	0.07
	NFC intake, kg/day	1.92^b^	2.31^a^	2.57^a^	0.041	0.024
	GE intake, MJ/day	100.8^b^	117.4^a^	124.9^a^	2.74	<0.01
	ADG, kg/day	1.10^b^	1.33^a^	1.36^a^	0.085	0.448
12 months						
	Age, month	11.7	11.6	12.4	0.72	0.477
	BW, kg	326.8^b^	336.0^b^	363.6^a^	10.27	0.036
	DM intake, kg/day	6.98^b^	7.06^b^	7.18^a^	0.233	0.001
	OM intake, kg/day	6.50^b^	6.56^b^	6.62^a^	0.227	0.017
	NDF intake, kg/day	2.57^a^	2.30^b^	2.10^b^	0.100	0.038
	NFC intake, kg/day	2.69^b^	2.96^b^	3.19^a^	0.106	0.001
	GE intake, MJ/day	115.9^b^	117.9^b^	124.6^a^	0.500	<0.01
	ADG, kg/day	0.97^b^	1.14^b^	1.39^a^	0.062	0.010
15 months						
	Age, month	14.7	14.6	14.9	0.33	0.965
	BW, kg	402.2^b^	424.4^a^	429.3^a^	8.82	0.945
	DM intake, kg/day	7.44^c^	7.78^b^	7.96^a^	0.234	0.014
	OM intake, kg/day	6.86^c^	7.22^b^	7.42^a^	0.126	<0.01
	NDF intake, kg/day	2.93^a^	2.53^b^	2.18^c^	0.228	0.031
	NFC intake, kg/day	3.06^b^	3.26^a^	3.30^a^	0.179	<0.01
	GE intake, MJ/day	124.9^c^	129.9^b^	132.2^a^	1.69	<0.01
	ADG, kg/day	0.87^b^	0.99^a^	1.05^a^	0.026	0.005

BW = body weight, OM = organic matter, NDF = neutral detergent fiber, ADF = acid detergent fiber, NFC = non-fibrous carbohydrate, GE = gross energy, ADG = average daily gain, C30 = diet containing 30% of concentrate, C40 = diet containing 40% of concentrate, C50 = diet containing 50% of concentrate, SEM = standard error of means. ^a,b,c^ values within a row with different superscripts differ significantly at *p* < 0.05.

**Table 3:** Effects of different dietary concentration level on apparent nutrient digestibility of Holstein cows at age of 9, 12 and 15 months.

Item		Treatments			SEM	*p-*Value
		C30	C40	C50		
9 months						
	Dry matter	73.3	74.8	75.3	1.00	0.710
	Organic matter	75.3	76.8	77.2	1.00	0.740
	Crude protein	65.6^c^	69.3^b^	76.5^a^	1.53	0.003
	Neutral detergent fiber	69.2^c^	72.4^b^	76.9^a^	1.52	0.012
	Acid detergent fiber	63.0^c^	69.4^b^	72.9^a^	1.87	0.041
12 months						
	Dry matter	73.5^b^	74.7^b^	78.3^a^	0.96	0.017
	Organic matter	77.1^b^	78.6^b^	81.9^a^	0.92	0.047
	Crude protein	65.6^b^	69.3^b^	76.5^a^	1.53	0.002
	Neutral detergent fiber	60.8^b^	65.4^a^	66.9^a^	1.66	0.029
	Acid detergent fiber	68.4	69.4	71.1	1.46	0.141
15 months						
	Dry matter	75.7^b^	76.5^b^	83.2^a^	1.04	<0.01
	Organic matter	77.9^b^	78.7^b^	84.9^a^	0.99	<0.01
	Crude protein	72.7^b^	72.2^b^	79.6^a^	1.10	<0.01
	Neutral detergent fiber	73.1^b^	75.4^a^	76.7^a^	0.92	0.028
	Acid detergent fiber	71.3^b^	73.5^b^	77.2^a^	1.11	0.046

C30 = diet containing 30% of concentrate; C40 = diet containing 40% of concentrate; C50 = diet containing 50% of concentrate, SEM = standard error of means. ^a,b,c^ values within a row with different superscripts differ significantly at *p* < 0.05.

**Table 4:** Effects of different dietary concentration level on enteric methane (CH_4_) emissions of Holstein cows at age of 9, 12 and 15 months.

Item		Treatments			SEM	*p-Value*
		C30	C40	C50		
9 months						
	CH_4_, g/day	114.90^a^	107.10^b^	93.66^c^	2.584	<0.01
	CH_4_/MBW, g/kg^0.75^	1.68^a^	1.59^b^	1.42^c^	0.031	0.002
	CH_4_/DM intake, g/kg	20.57^a^	16.56^b^	13.57^c^	0.643	<0.01
	CH_4_/OM intake, g/kg	26.15^a^	21.39^b^	17.15^c^	0.734	<0.01
	CH_4_/NDF intake, g/kg	60.66^a^	47.55^b^	35.40^c^	2.99	<0.01
	CH_4_-E, MJ/day	6.40^a^	5.96^b^	5.21^c^	0.131	<0.01
	CH_4_-E/GE intake	0.0686^a^	0.0552^b^	0.0454^c^	0.00264	<0.01
12 months						
	CH_4_, g/day	159.68^a^	133.16^b^	119.32^c^	5.054	<0.01
	CH_4_/MBW, gkg^0.75^	2.09^a^	1.71^b^	1.50^c^	0.079	0.001
	CH_4_/DM intake, g/kg	22.88^a^	18.85^b^	16.63^c^	0.903	<0.01
	CH_4_/OM intake, g/kg	27.13^a^	22.65^b^	20.40^c^	0.958	<0.01
	CH_4_/NDF intake, g/kg	63.77^a^	55.81^b^	54.60^b^	1.744	0.019
	CH_4_-E, MJ/day^-1^	7.89^a^	7.41^b^	6.64^c^	0.281	<0.001
	CH_4_-E/GE intake	0.0742^a^	0.0618^b^	0.0558^b^	0.00321	<0.001
15 months						
	CH_4_, g/day	219.58^a^	214.86^b^	193.77^c^	4.17	<0.01
	CH_4_/MBW, g/kg^0.75^	2.39^a^	2.26^a^	2.02^b^	0.058	0.013
	CH_4_/DM intake, g/kg	23.17^a^	19.94^b^	16.92^c^	0.776	<0.01
	CH_4_/OM intake, g/kg	24.95^a^	21.54^b^	18.20^c^	0.837	<0.01
	CH_4_/NDF intake, g/kg	69.39^a^	67.12^b^	64.83^c^	1.312	0.039
	CH_4_-E, MJ/day	12.77^a^	11.96^b^	10.78^c^	0.232	<0.01
	CH_4_-E/GE intake	0.0769^a^	0.0665^b^	0.0568^c^	0.00374	<0.01

MBW = metabolic body weight, DM = dry matter, OM = organic matter, NDF = neutral detergent fiber, CH_4_-E = methane energy, GE = gross energy, C30 = diet containing 30% of concentrate; C40 = diet containing 40% of concentrate; C50 = diet containing 50% of concentrate, SEM = standard error of means. ^a,b,c^ values within a row with different superscripts differ significantly at *p* < 0.05.

**Table 5:** Prediction equations of methane (CH_4_) emission for Holstein heifers at age of 9, 12, and 15 months.

Item	Equations	SE	R^2^	Eq.
CH_4_	= 0.13 _(0.106)_ × BW + 68.6 _(29.15)_	0.330	0.47	(1)
	= 24.21 _(1.133)_ × DM intake − 51.3 _(7.34)_	0.999	0.67	(2)
CH_4_-E	= 0.08 _(0.004)_ × GE intake − 2.72 _(0.467)_	0.999	0.69	(3)
CH_4_	= 0.19 _(0.151)_ × BW + 78.6 _(49.64)_	0.461	0.42	(4)
	= 36.27 _(6.712)_ × DM intake − 87.8 _(12.24)_	0.782	0.71	(5)
CH_4_-E	= 0.11 _(0.012)_ × GE intake − 4.65 _(1.785)_	0.766	0.72	(6)
CH_4_	= 0.29 _(0.161)_ × BW + 84.9 _(68.61)_	0.461	0.46	(7)
	= 51.72 _(4.640)_ × DM intake − 193.9 _(22.49)_	0.979	0.74	(8)
CH_4_-E	= 0.18 _(0.042)_ × GE intake − 9.70 _(2.071)_	0.973	0.67	(9)

CH_4_-E = methane energy (MJ/day), BW = body weight (kg), DM = dry matter (kg/day), GE = gross energy (MJ/day); SE = standard error

**Table 6:** Development of methane prediction models for Holstein heifers using the whole data sets.

Item^1^	Equations	SE	R^2^	Eq.
Linear models				
CH_4_-E (MJ/day)	0.026 (0.0043) × BW (kg) + 0.69 (0.431) × DM intake (kg/day) − 5.564 (1.1940)	0.337	0.742	(10)
	3.18 (0.408) × DM intake (kg/day) + 1.74 (0.598) × NDF intake (kg/day) − 9.426 (2.6003)	0.682	0.593	(11)
	1.75 (0.399) × DM intake (kg/d) - 2.71 (0.648) × NFC intake (kg/day) − 8.552 (2.4150)	0.549	0.655	(12)
	0.024 (0.0041) × BW (kg) + 1.22 (0.456) × DM intake (kg/day) + 1.15 (0.459) × NDF intake (kg/day) − 5.389 (2.0681)	0.261	0.777	(13)
	0.023 (0.0037) × BW (kg) + 0.28 (0.038) × DM intake (kg/day) − 2.04 (0.486) × NFC intake (kg/day) − 4.872 (1.8634)	0.132	0.820	(14)
Non-linear models				
CH_4_-E (MJ/day)	5.564 (1.1206) × exp (0.0276(0.0037) × DM intake (kg/day))	0.396	0.461	(15)
	4.333 (1.0177) × DM intake (kg/day) ^0.232(0.0452)^	0.601	0.446	(16)
	2.465 (0.7452) × exp (0.0075(0.0008) × NDF intake (kg/d))	0.452	0.411	(17)
	2.204 (0.6514) × NDF intake (kg/day) ^0.084 (0.0072)^	0.377	0.489	(18)
	0.926 (0.0452) × exp (0.0672(0.00121) × NFC intake (kg/day))	0.514	0.385	(19)
	0.527 (0.0271) × NDF intake (kg/day) ^0.541 (0.0362)^	0.602	0.434	(20)

^1^ CH_4_-E = methane energy, BW = body weight, DM = dry matter, NDF = neutral detergent fiber, NFC = non-fibrous carbohydrate; SE = standard error.
